# Effects of chronic unpredictable mild stress induced prenatal stress on neurodevelopment of neonates: Role of GSK-3β

**DOI:** 10.1038/s41598-018-38085-2

**Published:** 2019-02-04

**Authors:** Mahino Fatima, Saurabh Srivastav, Mir Hilal Ahmad, Amal Chandra Mondal

**Affiliations:** Cellular and Molecular Neurobiology Laboratory, School of Life Sciences, Jawaharlal Nehru University, New Delhi, 110067 India

## Abstract

Prenatal stress (PNS) has gained attention with regard to its impact on hippocampal neurogenesis in neonates which serves as a risk factor for postnatal neurodevelopmental deficits. Evidences from animal models have suggested that depression responsive hypothalamic-pituitary-adrenal (HPA) axis and its hormonal response via cortisol, is responsible for critical neurodevelopmental deficits in the offspring which is transduced due to gestational stress. But knowledge in the area of assessing the effects of maternal chronic unpredictable mild stress (CUMS) on neurogenesis and expression of some key signaling molecules in the offsprings are limited. We have used Wistar rats to induce PNS in offsprings by maternal CUMS during pregnancy. Prefrontal cortex (PFC) and hippocampus were assessed for biomarkers of oxidative stress, neurogenesis, neurodevelopmental signaling molecules and DNA damage in the male Wister offsprings. Our investigations resulted in sufficient evidences which prove how maternal psychological stress has widespread effect on the fetal outcomes via major physiological alteration in the antioxidant levels, neurogenesis, signaling molecules and DNA damage. PNS leads to the upregulation of GSK-3β which in turn inhibited mRNA and protein expressions of sonic hedgehog (SHH), β-catenin, Notch and brain derived neurotrophic factor (BDNF). The study explored multifaceted signaling molecules especially, GSK-3β responsible for crosstalks between different neurodevelopmental molecules like SHH, Notch, BDNF and β-catenin affecting neurodevelopment of the offsprings due to PNS.

## Introduction

Depression is an affective disorder with the incidence being 2–3 times more in women than in men^1^. Pregnancy period is the highest risk factor for women to develop depression^2^ therefore, fetus is exposed to PNS. PNS results in altered neurodevelopment in the fetus due to exposure of chronic stress to a pregnant mother owing to the environmental adversities, resulting from stressful life events^3^. Since, there is no direct neural circuit between the dams and the fetus but most of the fetal transduction takes place via maternal physiological signal through placenta, therefore, this prenatal period is critical for the developing fetus. PNS has gained attention with regard to both its impact on hippocampal neurogenesis in neonates^4,5^ and as a risk factor for altered postnatal neurodevelopment in the adult offspring^6^. In this regard, protocols for CUMS to generate preclinical animal model was initiated^7^. Recently improved CUMS for gestating dams has been adopted^8^ for animal models involving mild stressors. This type of chronic stress approach could be beneficial for the gestational studies on the behavioral and neurophysiological disorders stimulated by daily life environmental challenges during pregnancy^8,9^. Maternal stress and depression during pregnancy reprograms HPA axis, decrease placental barrier and increase glucocorticoid/cortisol level affecting neurodevelopment in the rat offsprings via signaling pathways. Altered epigenetic regulation and reprogramming of HPA axis altogether may have profound and compromising effects on fetal brain development across several generations^10^. Therefore, we have focused on the effects of PNS and chronic depression on the neurodevelopment and physiological outcomes of the offsprings through disturbances in the key developmental signaling molecules and neurotrophin level. Stress has profound effect on several cellular functions including plasma antioxidants levels as evidenced in depressive patients^11,12^. Oxidative stress plays an important mechanism in the pathophysiology of depression in rodents and humans^13,14^. Several studies reported that stress mediated alteration in the antioxidant enzymes, reactive oxygen species (ROS) and glutathione levels in the brain regions especially, PFC and hippocampus^15^ are most vulnerable to the pathophysiology of depression.

Stress mediates its deleterious effects on neurogenesis, leading to depletion in cell proliferation in the PFC and hippocampus, validated by bromodeoxyuridine (BrdU) cell proliferation assay^16^. Neurogenesis in the brain is also supported by BDNF, the most abundant neurotrophin, involved in neuronal survival, differentiation, and synaptic plasticity. Neurotrophin hypothesis of depression suggests alterations in BDNF level, implicated in the pathophysiology of cognitive disorders^17^ especially in major depressive disorders.

Although neurogenesis is characterized by embryonic brain but limited neurogenesis in adult hippocampus remain elusive and several signaling proteins found to be involved during this process. In this milieu, we found interaction of several proteins and support neurogenesis in the hippocampus of embryonic and adult brain which we tried to unravel through the possible crosstalks between these molecules. Studies have signified that SHH plays a critical role in many neurological diseases. It affects the responsiveness of neural progenitor cells (NPCs) and neurons to other signals, and prominent among these signals are Notch and BDNF^18,19^. This responsiveness of cells during interactions between SHH and Notch regulating neural cell plasticity may be qualitative or quantitative depending upon location of that cell within SHH concentration gradient. Notch signaling molecule plays important role in development is found to be expressed in subgranular zone (SGZ) and subventricular zone (SVZ) of postnatal mouse brain^20^.

Another hallmark gene in depression is GSK-3β which is widely expressed in the brain and is inhibited by phosphorylation. GSK-3β regulates hippocampal neurogenesis and neuroprotection. It is the intersection of several signaling pathways that regulates downstream targets relevant to depressive disorders^21,22^. β-catenin is one of the primary downstream target of GSK-3β, which when phosphorylated is targeted for proteasomal degradation^23^. GSK-3β inhibition reduces this degradation and increases β-catenin availability for structural support of cell and regulation of gene transcription, depending upon its cellular location. The aim of the present study is to evaluate the effect of PNS on oxidative stress and neurogenesis in the neonatal brain via important neurodevelopmental signaling pathways, neurotrophin production and DNA damage.

## Results

### Validation of stress induction in dams

**Figure 1:**
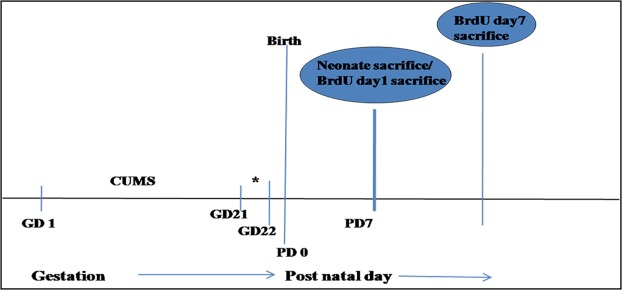
Timeline of experiment showing CUMS administration between GD1-21. For prenatal studies, pups sacrificed on PD7. *Mothers were subjected to behavioral tests.

Validations of stress induction in dams were assessed by two behavioural tests and maternal serum corticosterone level. Figure 1 shows timeline of the experimental procedures used in the current study.

#### Sucrose preference test

**Figure 2:**
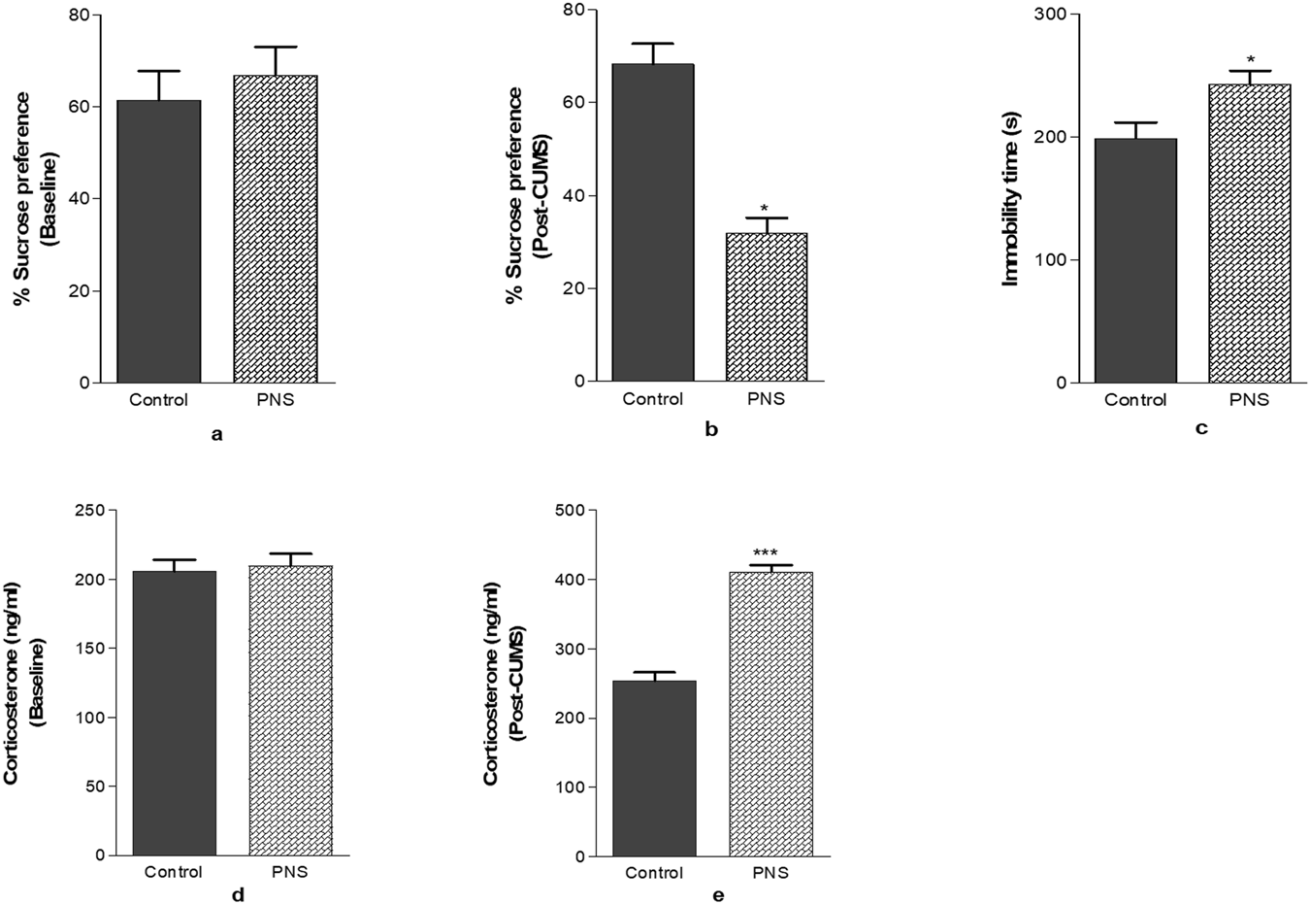
Validation of CUMS-administration to the dams using behavioral tests and serum corticosterone levels. Bar graph showing changes in behavior at (**a**) baseline and (**b**) post-CUMS period in sucrose preference test and (**c**) immobility time in the forced swim test and serum corticosterone levels at (**d**) baseline and (**e**) post-CUMS in rats after CUMS period. Significance difference is indicated by *p < 0.05, ***p < 0.001 when compared to the control. Each value is represented as mean ± SEM (n = 6).

Figure 2(a,b) displays SPT in the control and the PNS group at the baseline and after CUMS-administration. Student’s t-test revealed no significant difference for SPT in the control and the PNS groups which were carried out on 1 day before the beginning of the CUMS protocol. Test performed at the end of CUMS protocol showed significant (p < 0.05) reduction in the sucrose preference in the PNS group.

#### Forced swim test

Figure 2(c) shows the effect of CUMS-administration on immobility time in FST in rats performed after CUMS period. Student’s t-test revealed significant increase (p < 0.05) in immobility time of the PNS group when compared to the control.

#### Corticosterone level in dams

Figure 2(d,e) displays corticosterone level in the control and the PNS group at the baseline and after CUMS-administration. Student’s t-test revealed no significant difference of corticosterone levels in the control and the PNS groups, carried out on 1 day before the beginning of the CUMS protocol. Test performed at the end of the CUMS protocol showed highly significant (p < 0.001) elevation of the corticosterone level in the PNS group.

### Effect of CUMS induced PNS on changes in the body weight of pups

Table 1 summarizes data on the litter size, number of males and females born, body weights and changes in the body weight. PNS pups showed lower change in the body weight compared to the pups born to nonstressed control mothers but Student’s t-test showed that changes in the body weight were not statistically significant.

**Table 1:** Summarized data on the litter size, number of males and females born, body weights and changes in body weight.

Groups of dams (n = 6)	Litter size (Mean ± SD)	Minimum-maximum (No. of pups born)	No. of males and females	Body weight of pups (PD = 0 and PD = 7)		Changes in body weight
Control	12 ± 1.26	10–13	♂ = 6 ± 0.63 ♀ = 6 ± 0.83	PD = 0 PD = 7	11.32 ± 1.83 19.56 ± 2.08	8.17 ± 0.24
PNS	12.83 ± 1.16	11–14	♂ = 6 ± 0.63 ♀ = 7 ± 0.89	PD = 0 PD = 7	10.18 ± 0.72 17.90 ± 0.50	7.55 ± 0.40

### Effect of CUMS induced PNS on the biomarkers of oxidative stress

**Figure 3:**
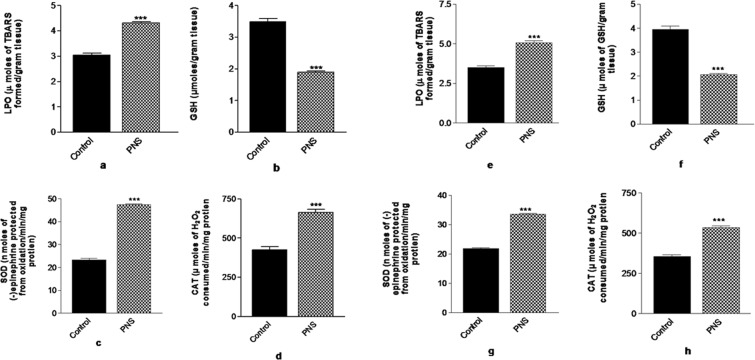
Effect of CUMS treatment on (**a**) LPO (**b**) GSH content (**c**) SOD activity and (**d**) CAT activity in the PFC and (**e**) LPO (**f**) GSH content (**g**) SOD activity and (**h**) CAT activity in the hippocampus of neonates. Significant difference is indicated by ***p < 0.001 when compared to the control. Each value is represented as mean ± SEM (n = 6).

Figure 3 depicts the effect of CUMS treatment on LPO, GSH content, SOD activity and CAT activity in the PFC and hippocampus of the neonatal rats. Student’s t-test revealed significant increase (p < 0.001) of LPO in the PFC for PNS rats (Fig. 3a). GSH levels were reduced significantly in the PFC (p < 0.001) for PNS rats compared to the control rats (Fig. 3b) in PFC. SOD (Fig. 3c) and CAT (Fig. 3d), the major antioxidant enzymes levels were significantly elevated (p < 0.001) with the induction of stressors to the rats during prenatal period.

Student’s t-test revealed significant increase (p < 0.001) of LPO in the hippocampus for PNS rats (Fig. 3e). GSH levels were reduced significantly (p < 0.001) in the hippocampus of PNS rats compared to the control rats (Fig. 3f). SOD (Fig. 3g) and CAT (Fig. 3h), the major antioxidant enzymes levels were significantly elevated (p < 0.001) with the induction of stress to the rats during their prenatal period.

### Effect of CUMS induced PNS on the proliferation and differentiation of neurons

**Figure 4:**
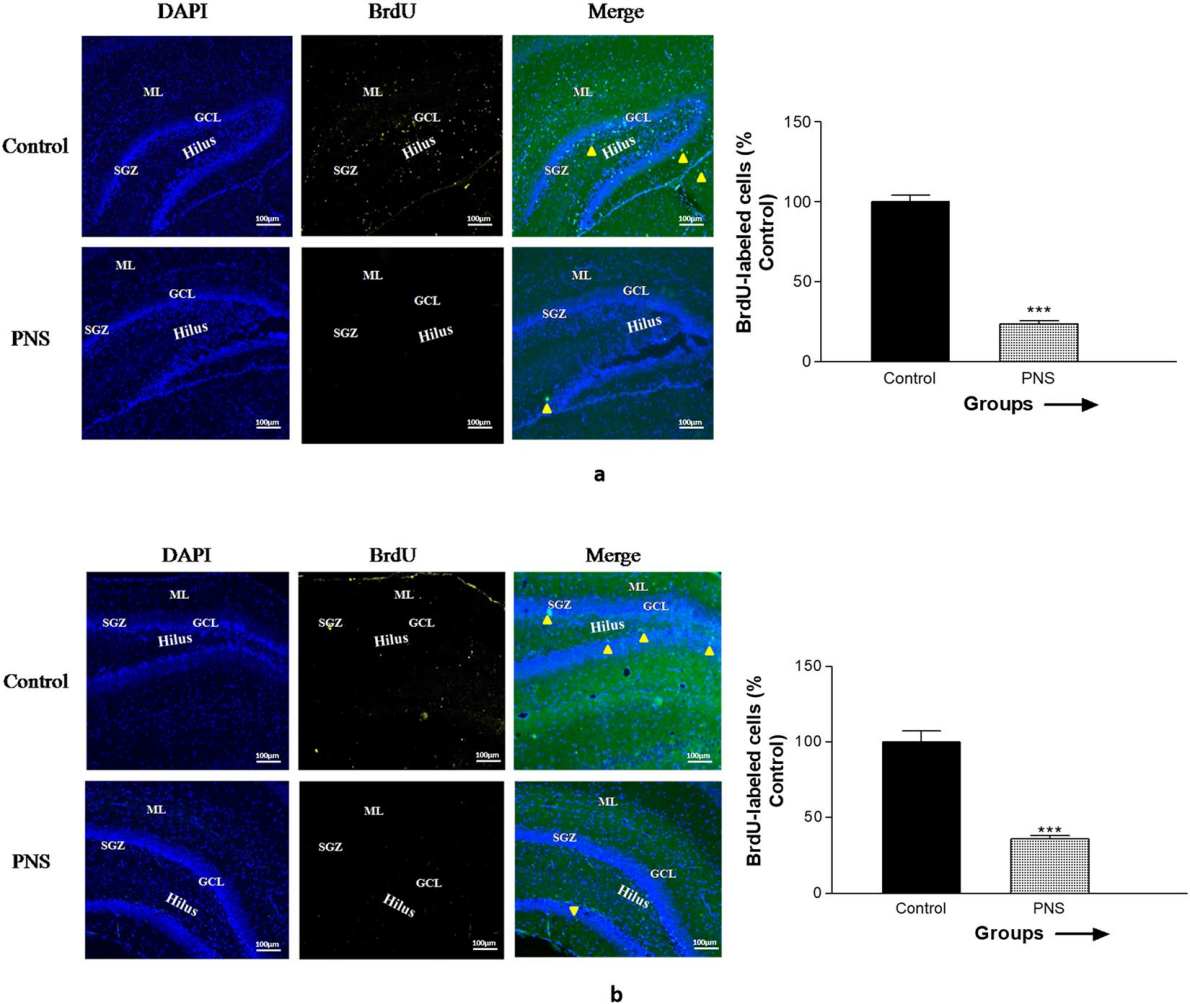
Effect of CUMS treatment on (**a**) hippocampal cell proliferation in the dentate gyrus after 24 h of BrdU injection and (**b**) hippocampal cell differentiation in the dentate gyrus after 7 days of BrdU injection in 7 days old pups. Quantitative data showing the effect of PNS on the number of BrdU-positive cells proliferated in the dentate gyrus. Representative images showing immunostaining of BrdU (Green) positive cells proliferated/differentiated with DAPI (Blue) counterstaining in dentate gyrus following PNS treatment. Merge figures showing yellow arrow heads point to BrdU labeled positive cells found in the dentate gyrus. ML, molecular layer; GCL, granular cell layer; SGZ, subgranular zone (Scale bars: 100 µm). Bar graph showing significant difference is indicated by ***p < 0.0005 when compared to the control. Each value is represented as mean ± SEM.

Figure 4 reveals the effect of CUMS treatment on the hippocampal cell proliferation in the DG of 7 days old pups and cell differentiation in the DG of 14 days old pups. BrdU was administered 24 h before the fixation of brain for locating proliferated cells (Fig. 4a). BrdU- labeled cells were located near SGZ of DG. BrdU incorporation into new cells were significantly decreased (p < 0.001) in prenatally stressed rats compared to the non-stressed control rats.

For locating differentiated neurons, we incorporated BrdU and examined BrdU-positive cells after 7 days of BrdU incorporation (Fig. 4b). Quantitative data showed significant decrease (p < 0.001) in number of BrdU-positive cells proliferated in the DG of the PNS rats compared to the control rats. Representative images showing immunostaining of BrdU (Green) positive cells proliferated/differentiated with DAPI (Blue) counterstaining in the DG following PNS treatment. Merge figures showing yellow arrow heads point to BrdU labeled positive cells found in the DG.

### Effect of CUMS induced PNS on the developmental genes expression

**Figure 5:**
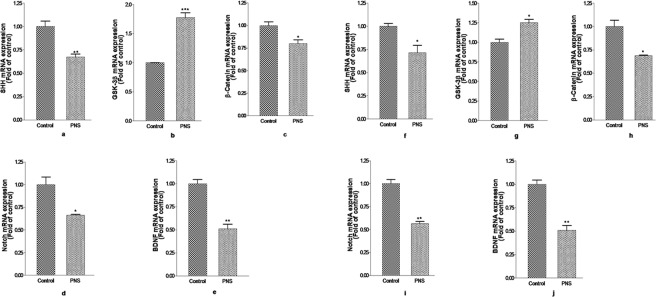
Effect of CUMS treatment on mRNA expression of (**a**) SHH, (**b**) GSK-3β (**c**) β-catenin (**d**) Notch and (**e**) BDNF in the PFC and (**f**) SHH, (**g**) GSK-3β (**h**) β-catenin (**i**) Notch and (**j**) BDNF in the hippocampus of neonates. Significant difference is indicated by *p < 0.05; **p < 0.01 and ***p < 0.001 when compared to the control. Each value is represented as mean ± SEM (n = 3).

Figure 5 demonstrates the effect of CUMS treatment on the mRNA expression of Shh, Gsk-3β, β-catenin, Notch and Bdnf in the PFC and hippocampus of the neonatal rats. PNS rat shows significantly lower (p < 0.01) Shh expression (Fig. 5a) but significantly higher (p < 0.001) expression of Gsk-3β (Fig. 5b) in the PFC region of the brain. Expression of β-catenin (Fig. 5c) and Notch (Fig. 5d) was also reduced significantly (p < 0.05) in the PFC of the PNS rats compared to the non-stressed control rats. Bdnf expression (Fig. 5e) reduced significantly (p < 0.01) in the PFC region in the brain of stressed rats.

Student’s t-test revealed significant decline (p < 0.05) in SHH expression (Fig. 5f) while an increase (p < 0.05) in the mRNA expression of GSK-3β (Fig. 5g) in the hippocampus of rat brain. There were significant reduction in β-catenin (p < 0.05) (Fig. 5h), Notch (p < 0.01) (Fig. 5i) and BDNF (p < 0.01) (Fig. 5j) mRNA expression in the hippocampus of rats exposed to PNS than non-stressed controls.

### Effect of CUMS induced PNS on the protein expression of SHH, GSK-3β, β-catenin, Notch and BDNF in the PFC and Hippocampus of PNS rat brain

**Figure 6:**
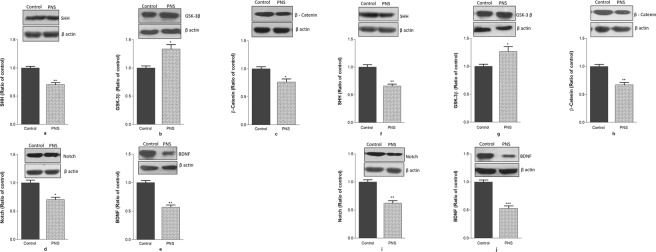
Representative Western blot and summary bar graph showing the effect of prenatal stress on the expression of (**a**) SHH (**b**) GSK-3β (**c**) β- catenin (**d**) Notch and (**e**) BDNF in the PFC and (**f**) SHH, (**g**) GSK-3β (**h**) β-catenin (**i**) Notch and (**j**) BDNF in the hippocampus of PNS rat brain. Significance difference is indicated by *p < 0.05,**p < 0.01 and ***p < 0.001 when compared with the control. Each value is represented as mean ± SEM. The display of cropped blots is shown to improve the clarity and conciseness of the presentation. The full-length blots are presented in Supplementary Figures 1 and 2.

Figure 6 shows western blot and corresponding bar graph representing expression of SHH, GSK-3β, β-catenin, Notch and BDNF (relative density of the bands) for the effect of PNS administration in the PFC and hippocampus of rat brain. PNS rats show significantly lower (p < 0.01) SHH expression (Fig. 6a) but significantly higher (p < 0.05) expression of GSK-3β (Fig. 6b) in the PFC region of the brain. Expression of β-catenin (Fig. 6c) and Notch (Fig. 6d) were also reduced significantly (p < 0.05) in the PFC of the PNS rats compared to the non-stressed control rats. BDNF expression reduced significantly (p < 0.01) in the PFC region in the brain of stressed rats.

Student’s t-test revealed significant decline (p < 0.01) in SHH expression (Fig. 6f) while an increase (p < 0.05) in the protein expression of GSK-3β (Fig. 6g) in the hippocampus of the rat brain. There were significant reduction in β-catenin (p < 0.01) (Fig. 6h), Notch (p < 0.01) (Fig. 6i) and BDNF (p < 0.001) (Fig. 6j) protein expression in the hippocampus of rats exposed to PNS than non-stressed controls.

### Effect of CUMS induced PNS on DNA damage

**Figure 7:**
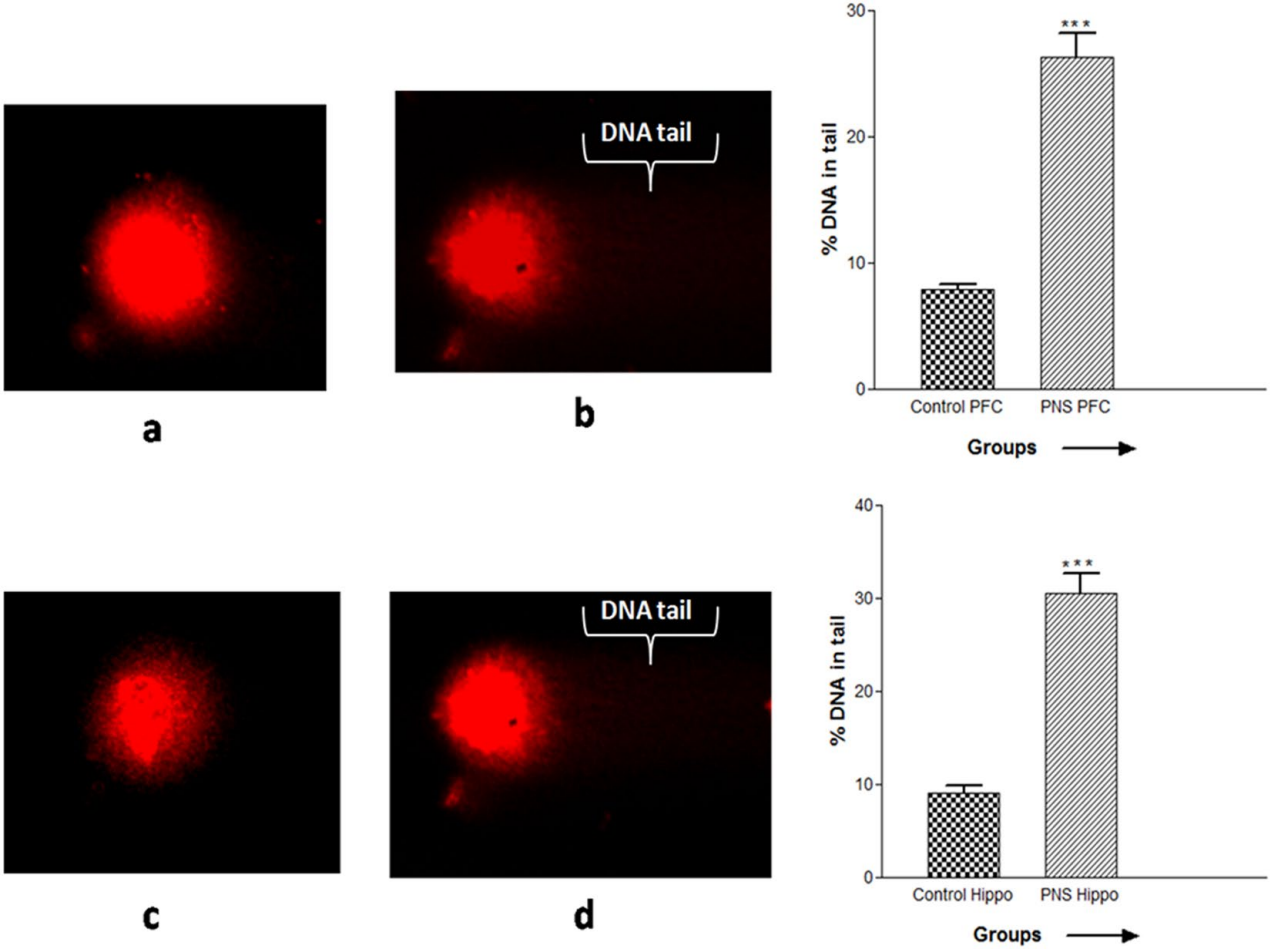
Representative images of comet assay in the PFC of (**a**) the control and (**b**) the CUMS-treated rats and in the hippocampus of (**c**) the control and (**d**) the CUMS-treated rats. Values in the bar graph represented as mean ± SEM (n = 6). Significant difference is indicated by ***p < 0.001 when compared to the controls.

Figure 7 reveals % tail DNA damage using bar graph and representative images showing comet in PFC and hippocampus of the control and the CUMS-treated rat’s offspring. Student’s t-test demonstrated significant (p < 0.001) increase in DNA content in comet tail in the PFC and hippocampal cells of the PNS rats when compared to the non-stressed control rats.

## Discussion

With the increasing burden of depressive disorders and behavioral abnormalities, it is important to understand early life exposures and underlying clinical aspects in detail. This is a major cause of morbidity and mortality adversely affecting PFC and hippocampus^24^ via deficit in BDNF signaling, impaired neurogenesis and loss of synapses^25^. Depression being multifactorial disorder initiated by both the genetic and environmental risk factors, provide an urgent need to study implicated pathways to give new insights into the understanding of the problem and the development of more efficacious medication. In this context, we have investigated the effect of CUMS induced PNS on neurodevelopmental aspects of neonates which may form the basis of behavioral and clinical implications in the later stages of life. Owing to complex nature of the depressive disorder, potential crosstalk between signaling pathways explored through which different pathological mechanism converge to affect neurogenesis in the neonates and eventually precipitate in depression.

Exposure to chronic mild stressors in unpredictable manner induced marked behavioral disturbances in dams which led to the development of PNS in the offspring rats. CUMS induced male PNS rats are used for its good etiological and predictive validity since, exposure to maternal CUMS induces a series of persistent neurobiochemical, and neuroendocrinal changes in the offsprings paralleling those observed in human depression^26^. Therefore, the CUMS induced model is one of the best validated animal models of depression for preclinical evaluation^27,28^. Our findings show that PNS mediates its effect through compromised neurogenesis which is in line with the other researchers^4,29,30^. Depression in pregnant rat which was validated by decreased sucrose consumption in SPT^31,32^ and an increased immobility time in FST^33^, characterized by anhedonia and behavioral despair^34,35^.

Results revealed that serum corticosterone level was highly increased in the stressed dams. During pregnancy, maternal glucocorticoids have direct effects on the fetal hippocampal neurons under normal physiological and chronic stress conditions. Earlier literatures have shown that HPA axis and its hormonal response via cortisol, is responsible for critical neurodevelopmental deficits in the offspring, transduced during gestational period. This leads to the alteration in feedback regulation of HPA axis due to down-regulation of hippocampal, hypothalamic, pituitary and adrenal gland receptors^36^. Development and maturation of HPA axis is species specific and depends on glucocorticoid exposure. Adrenal gland synthesizes cortisol that plays crucial role in the fetal neurodevelopment. Reprogrammed HPA axis has long life behavioural and functional adversities in the offsprings^10^. CUMS induced PNS in pups resulted in their lower body weights than the pups born to non-stressed control mothers but change was not statistically significant. PNS offsprings were exposed to higher levels of corticosterone during gestation which might have affected their weight gain. PNS pups appear to be born with altered metabolism that resulted in lower weight gains than the control pups.

Depression developed in the dams through CUMS is mediated through elevated ROS level in the PFC and hippocampus^37^ which leads to cascade of implicated physiological pathways in developing fetus. Since fetus is transduced to prenatal stress via maternal functions, so we assessed levels of oxidative stress in the PFC and hippocampus of the pups. Elevated ROS is thought to be an important mechanism in the pathophysiology of depression in rodents and humans^13,14,35^. Results with increased levels of lipid peroxidation, decreased GSH content and increased activity of SOD and CAT antioxidant enzymes are indicative of oxidative state of PFC and hippocampus of the pups. PFC and hippocampus are major brain structures involved in the pathophysiology of depression and CUMS induced oxidative stress^37^. Increased oxidative stress interferes with the intracellular cell signaling pathways^14^ and increase redox signaling^38^ that consequently lead to mood disorders.

Effects of PNS and depression on offspring neurogenesis and physiological outcomes, is limited and our findings showed dysregulation in signaling pathways and BDNF profile. Rodent models have been shown to translate PNS in the male offsprings via maternal depression similar to humans^39,40^. These perspectives provide novel insights into functional programming of normal states. In this regard, we focused on the hippocampal neurogenesis, responsible for maintaining brain plasticity. NPCs generate glial cells or neurons which predominantly occur in the DG of the hippocampus, the SVZ and very small numbers permanently integrate into the adult brain circuits. This important role of hippocampal neurogenesis maintains brain plasticity and learning process. The fate of NPCs are regulated by stimuli provided by growth factors, Wnt, β-catenin, Notch, SHH which monitor intracellular signaling pathways and transcriptional factors^41^. β-catenin cytoplasmic protein plays a crucial role in the canonical Wnt signaling pathway, the stability of which is regulated by the APC/Axin/CK1/GSK3β destruction complex. Presence of the Wnt ligand is essential for the stabilization of β-catenin and its translocation to the nucleus, where it interacts with the transcripts TCF/LEF to activate target gene expression. Thus, an abnormal Wnt/β-catenin signaling pathway has been associated with the pathophysiology of various neuropsychiatric disorders^42^. SHH, another crucial signaling molecule is required for an early tissue patterning and axon growth during brain development, affecting neuroplasticity^43,44^. In this milieu, we have found that mRNA expression of SHH in the PFC and hippocampus of stressed pups is decreased which is in line with the other findings^43,44^. Dysregulated expression of SHH is shown by various researchers leading to compromised neurodevelopment^45–48^. SHH affects responsiveness of NPCs and neurons to other signaling molecules majorly, Notch and BDNF regulating hippocampal neurogenesis and neuronal plasticity^18,19,49^.

Our findings have shown that maternal CUMS leads to the down regulation of mRNA expressions and protein levels of some key signaling molecules like SHH, β-catenin, Notch and BDNF which compromised neurogenesis in the PNS male offsprings. Study explored that the level of multifaceted signaling molecules GSK-3β is up-regulated which is responsible for crosstalks between different neurodevelopmental molecules like SHH, β-catenin, Notch and BDNF affecting neurodevelopment of the male offsprings due to PNS. GSK-3β identified as a regulator of cellular functions such as cell proliferation, stem cell renewal, and apoptosis. It is found in variety of tissues but highest levels found in the brain^50,51^. Evidences shown that dysregulation of GSK-3β inhibits SHH, Wnt/β-catenin and Notch signaling pathway^52,53^. Conditional GSK-3β over-expression in the forebrain results impaired memory and spatial learning in mice^54^. Over-expression of GSK-3β increased neuronal cell death, astrocytosis, gliosis and reduced LTP which was restored by reducing GSK-3β level to normal either by silencing the transgene or by treatment with lithium^55,56^. GSK-3β antagonises SHH signaling pathway by mediating the degradation of Gli proteins^57^. Cross-talk between the GSK-3β and Notch is also seen as GSK-3β binds and phosphorylate the intracellular domain of Notch, stabilizing the protein by reducing its degradation by the proteasome^58^. Interactions between Notch and other cell developmental pathways involving GSK-3β are involved in deciding fate of NPCs^41^. Transcriptional activity of Notch was enhanced by β-catenin^59^. β-catenin further controls progenitor cell proliferation in the regions of the developing brain^60^.Thus, these molecules are involved in critical cross-talks regulating neurogenesis during neuropsychiatric apathy. Our findings also corroborates with other studies which demonstrated that neuronal deficits in response to stress may be attributed to the dysfunction of GSK-3β/β-catenin (or Wnt/β-catenin) signaling mediated by increased glucocorticoid signalling^42,61^. Neurodevelopmental signaling pathways are studied here to unravel molecular mechanism in the pathophysiology of depression in neonates that might occur in adult life too, since some of the neurodevelopmental pathways are conserved in adult. Multifacet developmental signaling molecules can thus be used as efficacious drug targets in the depressive therapies.

In conclusion, studies using animal models relate neonatal neurogenesis as key aspect of the mechanism through which CUMS mediates its affects during initial neurogenesis, same mechanism may follow in the adult depressive disorders too as these developmental pathways are conserved in adult brain part especially hippocampus. SHH controls patterning and responsiveness of NPCs to Notch and BDNF signals and helps in neurogenesis during development, therefore, can be a potential therapeutic target for antidepressant action. Findings from the present study also indicate that stress may negatively affect neurogenesis through GSK-3β mediated inhibitory crosstalks of key developmental signaling genes and overall lowering of BDNF gene expression. It is possible that BDNF signaling modulates these signaling and thus sustains neurogenesis while GSK-3β regulates centrally to the effects of chronic stress on neonatal neurogenesis by crosstalks with other signaling pathways. It would be beneficial to take this into consideration when designing therapeutic targets which may increase the efficacy of drug by testing modulation of multiple target genes, suggesting stimulation of SHH while inhibition of GSK-3β. Scientific knowledge in the area of epigenetic processes like DNA methylation, histone acetylation and regulation of microRNA during prenatally stressed fetal neurodevelopment has also to be targeted with properly defined controls.

## Experimental Procedures

### Ethical statement for animal handling

All the experiments were carried out in accordance with the relevant guidelines, regulations and standard protocols were followed for animal handling and experimentation. Present study was carried on Wistar rat and all the experimental procedures were conducted in accordance with standard guidelines of CPCSEA, New Delhi after approval of Institutional Animal Ethics Committee (IAEC) Jawaharlal Nehru University, New Delhi (10/GO/ReBi/S/99/CPCSEA/10.03.1999).

### Experimental design and behavioral assessment

#### Animals and breeding

Adult virgin Wistar rats weighing 220–280 grams were housed in cages and were allowed to access food and water *ad libitum* unless otherwise indicated. All the rats were maintained on a constant 12 h light/dark cycle with constant temperature (21 ± 2 °C) and humidity (55%). Figure 1 shows timeline of the experimental procedures used in the current study. For breeding, 03 females were paired with 01 male per cage until appearance of vaginal plug, the birthday of pups was considered as gestation day (GD) 1. On GD 1, pregnant females were randomly assigned to control (C) and PNS groups and individually housed in plastic cages.

#### Induction of stress

PNS group of rats were subjected to CUMS procedure from GD1 to GD21, as previously described Cabrera (1999) and Biala (2001)^62,63^ with slight modifications in the procedure. Briefly, the CUMS paradigm comprising a variety of mild stressors was applied in an unpredictable manner, as listed: (1) soiled bedding (~150 ml water per cage) for 22 h; (2) one overnight period of permanent light; (3) cage tilting (~45°) for 22 h; (4) crowded housing for 12 h; (5) restraint stress for 6 h (3 sessions of 2 h with 30 min gap); (6) one overnight period of difficult access to food; (7) reversed light/dark cycle during the weekend. These stressors were given subsequently for 21 days and rats received one stressor everyday that makes each stressor unpredictable for the total course of three weeks of CUMS period. Controls were discreted from the CUMS group. Establishment of CUMS model of depression in pregnant female rats were validated by two behavioral tests viz. sucrose preference test (SPT) and force swim test (FST) for behavioral despair on GD 22. The day offspring were born, the pups were assigned as postnatal day (PD) = 0 and pup gender and individual body weights were determined. Litters of 8–13 pups with equal numbers of males and females were kept for the study, all other litters been culled. For each set of experiments, a maximum of two male pups were used from each litter to prevent any litter effects^64^. The pups were kept with their biological mothers for seven days as in rats considerable amount of neural development occur after birth^65^ and body weights were determined. Table 1 summarizes data on the litter size, number of males and females born, body weights and changes in body weight.

#### Sucrose Preference Test (SPT)

Animals were acclimatized to consume 1% (w/v) sucrose solution before 24 h of CUMS schedule using two-bottle free- choice method^66^. This training consisted of exposure of 1% sucrose to rats for 24 h adaption to sucrose solution. During test, each animal was presented simultaneously to premeasured two bottles, one containing sucrose solution and other only tap water and fluid intake was monitored for 24 h period. Baseline preference test was performed on day 0 (baseline) that is before the CUMS procedure and on the last day of the CUMS schedule. Preference to sucrose solution over tap water reflects core symptom of depression. The sucrose preference was calculated as percent using following ratio: Sucrose preference (%) = sucrose consumption/(sucrose consumption + water consumption) × 100.

#### Forced Swim Test (FST)

This test was performed just after the CUMS as described previously by Borges Filho *et al*.^37^. Briefly, each rat was placed into a transparent polypropylene cylindrical vessel (45 cm height × 35 cm diameter) containing 40 cm of water at 25 °C so that there is no escape and animal is forced to swim. Animals were kept in the apparatus for 6 mins of session each and total time for which each animal remained immobile was recorded as immobility time by two trained observers (data averaged from both the observers). The immobility period of the animal reflects the state of helplessness and behavioral despair^67^.

### Blood sampling and Corticosterone analysis

After the FST, blood samples were collected (via cardiac puncture) from the control (n = 6) and the stressed (n = 6) dams, into the test tubes. Samples were allowed to clot undisturbed for 30 min and centrifuged at 2000X g for 15 min at room temperature (RT). Serum was frozen at −20 °C for determination of corticosterone by using ELISA kit (Cayman, USA), according to the manufacturer’s instructions. Absorbance was read at 412 nm and the concentration was expressed as ng/ml.

#### BrdU injection

Procedure was performed as described byJaako-Movits *et al*.^16^. Briefly, the control and the experimental pups (n = 6) were administered BrdU (Calbiochem, USA) intraperitoneally (i.p.) in a total dose of 300 mg/kg, the BrdU dose was divided into three portions, and each portion (100 mg/kg) was administered at an interval of 2 h to the 7 days pups and were sacrificed after 24 h of the last injection for proliferative studies. While for survival and differentiation studies, another group of rats were sacrificed after 7 days of the last BrdU injection and their brains were taken for immunohistochemical detection of the neuronal cell proliferation and differentiation studies.

### Collection of PFC and hippocampus from rat brain

All the parameters were assessed after neonates were sacrificed on PD 7^68^. The male offsprings were anesthetized by intravenous injection of Thiopentone sodium (100 mg per kg body weight) (Pentothal sodium, Abbott laboratories) and decapitated. Brains were removed from the skull and kept on ice. With the reference landmark of bregma, the PFC and hippocampus were dissected according to Paxinos (1988)^69^. Isolated PFC and hippocampus were flash frozen into liquid nitrogen and kept in −80 °C till further analysis. For neurogenesis study, BrdU was incorporated into hippocampus by injecting BrdU and later was detected for BrdU positive cells in dentate gyrus (DG) using immunohistochemistry. For, oxidative stress in PFC and hippocampus, oxidative stress enzymes such as superoxide dismutase (SOD), catalase (CAT), level of reduced glutathione (GSH) and lipid peroxidation (LPO) were determined by biochemical estimations. Tissues were also used for the detection of expression levels of mRNA of SHH, GSK-3β, β-catenin, Notch and BDNF by using quantitative real time polymerase chain reaction (qRT-PCR). DNA damage in the hippocampus and PFC was determined by comet assay.

### Biochemical assays

#### Preparation of homogenate and post-mitochondrial supernatant (PMS)

The tissues were homogenized in 0.1 M phosphate buffer, pH 7.4 to obtain 10% homogenates using a Potter-Elvehjem homogenizer giving 6–8 strokes at medium speed keeping sample under ice. Homogenates were subjected to centrifugation in refrigerated centrifuge at 10,000 rpm for 20 min at 4 °C. The resulting pellets are the primary mitochondrial pellets and the supernatants are 10% post mitochondrial supernatants kept in −20 °C till further analysis.

### Protein estimation

Protein content in various samples was estimated using the method of Lowry *et al*.^70^ with bovine serum albumin (BSA) as protein standard.

#### Determination of Lipid peroxidation (LPO)

LPO was determined by the method of Mihara and Uchiyama^71^, with slight modifications. Briefly, 0.25 ml of tissue PMS was mixed with 25 μl of 10 mM butylated hydroxytoluene (BHT). 3 ml phosphoric acid (1%) and 1 ml of 0.67% thiobarbituric acid (TBA) were added and reaction mixture was incubated at 95 °C for 1 h. The absorbance was measured at 535 nm. The level of LPO was expressed as micromoles of thiobarbituric acid reactive substance (TBARS) formed/h/g of tissue using a molar extinction coefficient of 1.56 × 10^5^ M^−1^ cm^−1^.

#### Estimation of Reduced glutathione (GSH)

Tissue GSH levels were estimated as total ‘acid soluble sulfhydryl’ concentrations colorimetrically at 480 nm using Ellman’s reagent (DTNB) according to the procedure modified byJollow *et al*.^72^ PMS was precipitated with sulphosalicylic acid (4.0%) in the ratio of 1:1. The samples were kept at 4 °C for 1 hour and then subjected to centrifugation at 4000 rpm for 15 min at 4 °C. The assay mixture contained 0.4 ml supernatant, 2.2 ml of 0.1 M sodium phosphate buffer (pH 7.4) and 0.4 ml of 10 mM DTNB making a total volume of 3 ml. The optical density (OD) of reaction product was read immediately at 412 nm on a spectrophotometer and results were calculated using molar extinction coefficient (ε) 1.36 × 10^4^ M^−1^ cm^−1^ and results were expressed as nmol GSH/g tissue. 2.6 ml phosphate buffer and 400 µl DTNB was taken as blank.

#### Determination of Superoxide dismutase (SOD) activity

SOD activity was assayed using method of Misra and Fridovich^73^. The assay mixture consisted of 0.8 ml glycine buffer (50 mM, pH 10.4), 0.2 ml of supernatant (prepared in glycine buffer) and 20 µl of 2% epinephrine in a final volume of 1.02 ml. SOD activity can be measured kinetically at 480 nm. The activity is measured indirectly by the oxidized product of epinephrine i.e. adrenochrome. SOD activity is expressed as nmol of (−) epinephrine protected from oxidation by the sample compared with the corresponding reading in the blank. The activity was calculated by using its ε 4.02 × 10^3^ M^−1^ cm^−1^and results were expressed as nmoles of epinephrine protected from oxidation/min/mg protein.

#### Estimation of Catalase (CAT) activity

CAT activity was estimated by using method of Claiborne, (1985)^74^ with slight modifications. 1.95 ml of 0.1 M phosphate buffer (pH 7.4) was taken and 1.0 ml of 0.05 M hydrogen peroxide and 50 µl PMS was added in a 3 ml cuvette. The total volume for the assay was 3 ml. OD was taken via kinetic method at 240 nm in a spectrophotometer. The activity was calculated by using its ε 39.6 M^−1^ cm^−1^ and expressed as µmoles of H_2_O_2_ consumed/min/mg protein.

### Immunohistochemistry

For BrdU immunohistochemistry, animals were deeply anesthetized by i.v.injection of Thiopentone sodium (100 mg/kg body weight) (Pentothal sodium, Abbott laboratories) and transcardially perfused with normal saline and then with 4% paraformaldehyde in phosphate buffered saline (PBS, 0.1 M, pH 7.4). After post fixation of 24 h of the brain in paraformaldehyde/PBS solution, microtome sections of 5 µm thick was cut coronally kept onto Poly-L-lysine coated glass slides and processed for immunohistochemistry (IHC). For IHC, tissue sections were rehydrated and treated with 0.1 M citrate buffer, pH 6.0 for 10 min in microwave oven and rinsed with PBS containing 0.05% Tween-20 (TBST). Sections were then incubated with 1 M HCl at 54 °C for 45 min followed by TBST washing. Sections were incubated with 0.3% H_2_O_2_ for 10 min and washed by TBST. Sections were incubated with 5% BSA in TBST, followed by overnight incubation with mouse monoclonal anti-BrdU antibody (CST, Bu20a) diluted in blocking buffer (CST, 1:200) at 4 °C. After being washed with TBST, sections were incubated with Alexa flour-conjugated goat anti-mouse (Abcam, ab150113) secondary antibody diluted in blocking buffer for 1 h at RT and section were counterstained with DAPI for 30 min.

### Image analysis

For cell counting, every sixth section throughout the hippocampus was considered for each rat brain. The number of BrdU positive cells in the GCL and SGZ of DG of each section was counted. BrdU-positive cells were visualized using FITC (green) and normal nucleus with DAPI (blue) filters.

### Western blotting

Following SDS-PAGE on 12% acrylamide gel, 50 µg proteins were resolved and transferred on PVDF membrane and blots were placed into the blocking solution (5% non-fat milk powder (w/v) in wash buffer) for 1 h at RT, rinsed briefly with wash buffer. Blot was incubated with the primary antibody (Rabbit anti SHH/anti GSK-3β/anti β-Catenin/anti Notch antibody, CST, 1:1000 dilutions; Rabbit anti BDNF antibody, Novus Biologicals, 1:1000 dilutions) diluted in the wash buffer at 4 °C for overnight. Blot was washed extensively in wash buffer (3 × 5 minutes) with gentle agitation. Anti-rabbit HRP-conjugated secondary antibody (CST) diluted (1:1000) in wash buffer was added and incubated for 1 h at room temperature with gentle agitation. The membrane was washed with gentle agitation as follows: 3 × 5 minutes in wash buffer. Protein bands were visualized by ECL methods for developing in X-ray film to detect protein signals. The expression of target protein was quantified by densitometry using ImageJ software and expression was normalized using β-actin as internal control. Data is represented as relative expression of target protein in PNS group to the control group.

### Quantitative real time polymerase chain reaction (qRT-PCR)

All the procedures were performed as described by Cheung *et al*.^75^. The mRNA sequences were obtained from NIH-GenBank and Specific primers were designed and purchased. Tissue samples were homogenized with Trizol. After incubation at RT chloroform was added. The samples were shaken vigorously and incubated at RT for 5 min. Samples were centrifuged for 15 min at 12000 rpm at 4 °C. The top layer was transferred to new tube containing Isopropanol and centrifuged again at 12000 rpm for 5 min. The pellet was dissolved in RNase free water and the total concentration of the RNA was determined using nanodrop for UV absorbance at 260 and 280 nm. The RNA was reverse transcribed with a reaction mixture containing 1 µg RNA using cDNA synthesis kit (Thermo Scientific). The total volume of the reaction mixture was 20 µl. After mixing, the whole sample was incubated for 10 min at 30 °C followed by 20 min at 42 °C and 5 min at 99 °C. The obtained cDNA was further amplified by qPCR in 20 µl total volume mix, using PCR mastermix (PowerSYBR Green PCR MasterMix, Thermo Fisher Scientific). The reaction substrates were mixed gently and subjected to qPCR for amplification. Selected genes and their primer sequences are given in the Table No. 2. Gene expression analysis was performed with 7500 Fast Real-time system (Applied Biosytems) and conditions for the reaction involved 40 cycles in a fixed sequence of 95 °C for 30 s, 62 °C for 15 s and 72 °C for 15 s. Gene expression was normalized using Gapdh as reference gene. Software used for analysis was 7500 Software v2.0.5 (Applied Biosystems).

**Table 2:** Sequences of forward and reverse primers for qRT-PCR. The primers were designed using NCBI portal (https://www.ncbi.nlm.nih.gov/).

Gene	Forward primer	Reverse primer
Sonic hedgehog (Shh)	CATCCCTTGGGAATGGCAGT	TGCTTATCTGGCAGTCGCTT
Glycogen synthase kinase 3 beta (Gsk-3β)	ACTCTACCTGAACAGCCCCA	AACGTGACCAGTGTTGCTGA
Beta catenin (β-catenin)	GGCTAACATTCGCCAGTGGA	TGCCACGTCAGCTGGTATAG
Notch	TTGGTCCGAGGGCATCTCTA	ACAGAGCTTGGGAACGGAAG
Brain-derived neurotrophic factor (Bdnf)	AGGGAAATCTCCTGAGCCGA	TAATCCAATTTGCACGCCGC
Glyceraldehyde-3-phosphate dehydrogenase (Gapdh)	AGTGCCAGCCTCGTCTCATA	GGTAACCAGGCGTCCGATAC

### Preparation of single cell suspension for comet assay

DNA damage was assessed using alkaline comet assay as described earlier^76^ with slight modifications^77^. To avoid additional DNA damage, all the steps were performed under the dim light condition. Freshly collected PFC and hippocampus samples were washed with chilled phosphate buffered saline-calcium magnesium free (PBS-CMF). Tissues were rinsed with PBS-CMF, 20 mM EDTA and 10% dimethyl sulphoxide (DMSO), pH 7.4 and filtered through 100 µm mesh strainer to get cell suspension, followed by centrifugation at 2000 rpm at 4 °C for 10 min. Cell pellet was collected and cell viability test was performed using Trypan blue exclusion method^78^ and samples showing viability more than 80 percent were preceded for comet assay. Cell suspensions were suspended in 0.5% low melting point agarose (LMPA) and overlaid on slides precoated with a fine layer of 1.25% normal melting point agarose (NMPA). Third layer of 0.75% LMPA was poured onto slides. Slides were immersed in lysing solution (pH 10) for 1 h at 4 °C to lyse cells. Then slides were immersed in chill electrophoresis buffer (pH > 13) for 20 min to allow DNA unwinding followed by electrophoresis at 25 V and 300 mA current in the same buffer for 30 min. After electrophoresis slides were neutralized with neutralizing buffer (pH 7.5).

Slides were dried and stained with ethidium bromide. Photographs were obtained at 400X. Six animals per group were analyzed and 50 cells per animal were scored randomly and analyzed with Cometscore^TM^software (version 1.5, TriTek Corporation, Sumerduck). Degree of DNA damage was represented as percent DNA in tail.

### Statistical analysis

The results are expressed as mean ± SEM. Statistical analysis was performed using GraphPad Prism software (version 5). Student’s t-test was performed to compare significant difference between the control and PNS group. Significant difference is indicated by *p < 0.05; **p < 0.01 and ***p < 0.001 when compared to the controls.

## Supplementary information

Protein expression of SHH, GSK-3β, Notch, β-catenin and BDNF
