# Supplementary material for: Effects of chronic unpredictable mild stress induced prenatal stress on neurodevelopment of neonates: Role of GSK-3β
Source: Sci Rep. 2019 Feb 4;9:1305. doi: 10.1038/s41598-018-38085-2 (PMC6361942; doi:10.1038/s41598-018-38085-2)
Supplement: Supplementary file 1 — Protein expression of SHH, GSK-3β, Notch, β-catenin and BDNF [file 41598_2018_38085_MOESM1_ESM.docx]

**Effects of chronic unpredictable mild stress induced prenatal stress on neurodevelopment of neonates: Role of GSK-3β**

**Mahino Fatima, Saurabh Srivastav, Mir Hilal Ahmad, Amal Chandra Mondal***

Cellular and Molecular Neurobiology Lab, School of Life Sciences, Jawaharlal Nehru University, New Delhi, India-110067

*Corresponding author: E-mail: acmondal@mail.jnu.ac.in

Telephone: +91-11-2670-4505

**Supplementary information for figure 6**

The display of cropped blots is shown in figure 6 in the main manuscript to improve the clarity and conciseness of the presentation. The full-length blots are presented here as Supplementary Figure a, b, c, d & e.

**Supplementary Figure**


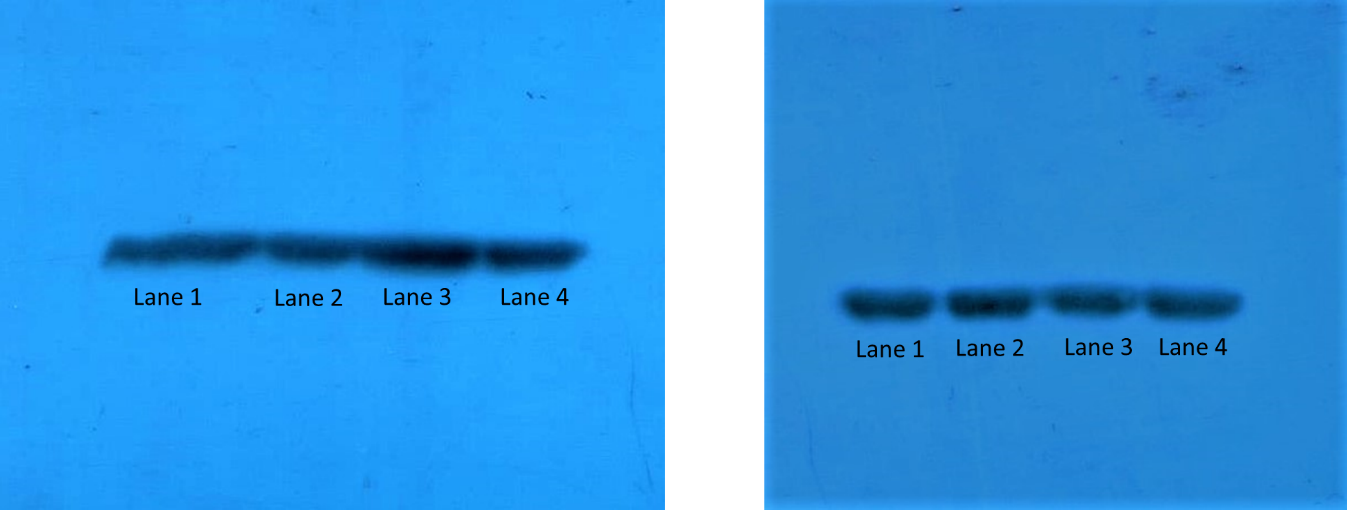


Lane 1: β-actin in control group in Prefrontal cortex (PFC)

Lane 2: β-actin in Prenatal stress (PNS) group in PFC

Lane 3: β-actin in control group in Hippocampus (Hippo)

Lane 4: β-actin in PNS group in Hippo

Lane 1: SHH in control group in Prefrontal cortex (PFC)

Lane 2: SHH in Prenatal stress (PNS) group in PFC

Lane 3: SHH in control group in Hippocampus (Hippo)

Lane 4: SHH in PNS group in Hippo

**a**


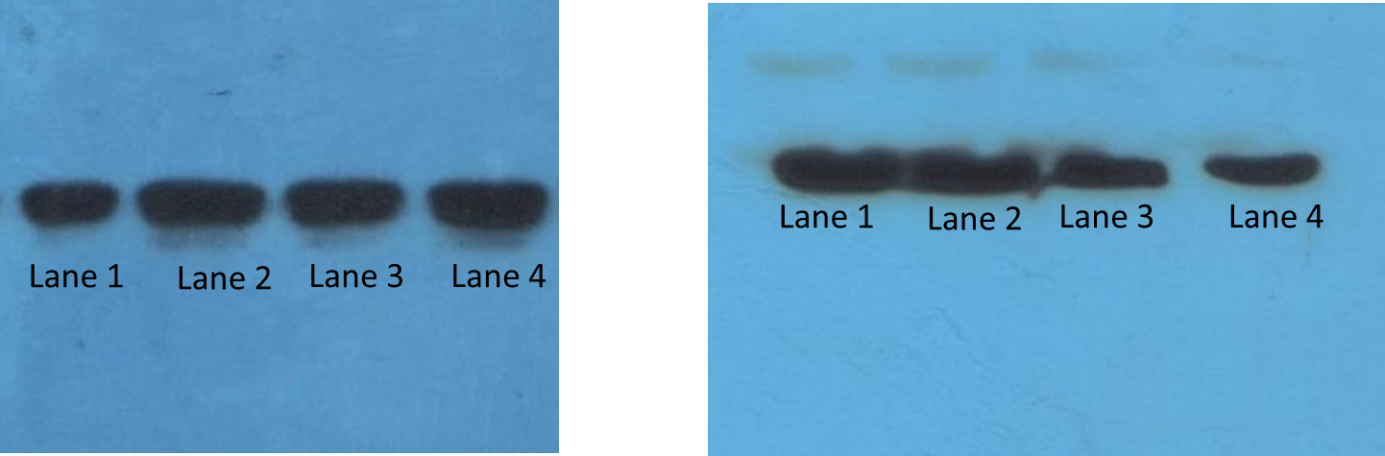


Lane 1: β-actin in control group in Prefrontal cortex (PFC)

Lane 2: β-actin in Prenatal stress (PNS) group in PFC

Lane 3: β-actin in control group in Hippocampus (Hippo)

Lane 4: β-actin in PNS group in Hippo

Lane 1: GSK-3β in control group in Prefrontal cortex (PFC)

Lane 2: GSK-3β in Prenatal stress (PNS) group in PFC

Lane 3: GSK-3β in control group in Hippocampus (Hippo)

Lane 4: GSK-3β in PNS group in Hippo

**b**


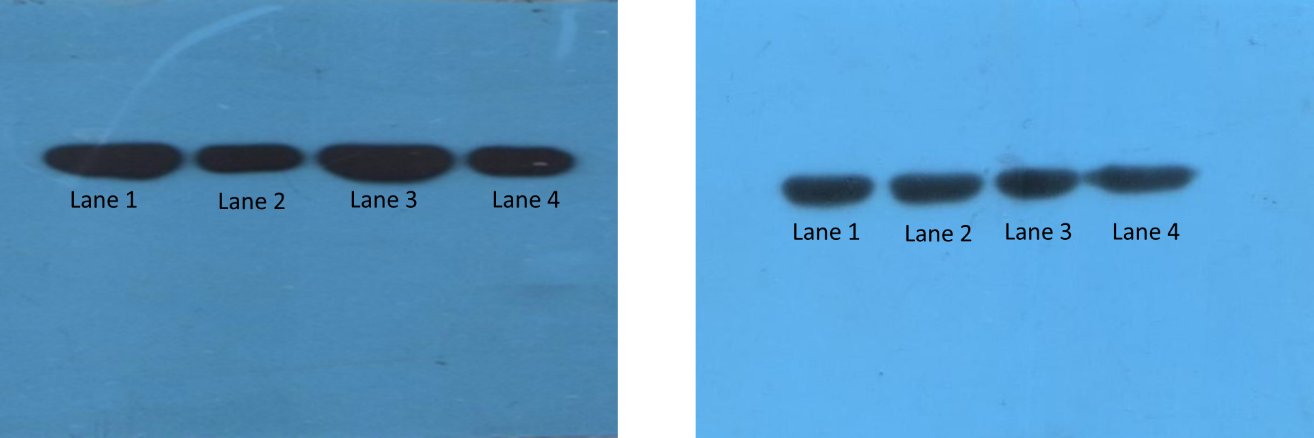


Lane 1: β-catenin in control group in Prefrontal cortex (PFC)

Lane 2: β-catenin in Prenatal stress (PNS) group in PFC

Lane 3: β-catenin in control group in Hippocampus (Hippo)

Lane 4: β-catenin in PNS group in Hippo

Lane 1: β-actin in control group in Prefrontal cortex (PFC)

Lane 2: β-actin in Prenatal stress (PNS) group in PFC

Lane 3: β-actin in control group in Hippocampus (Hippo)

Lane 4: β-actin in PNS group in Hippo

**c**


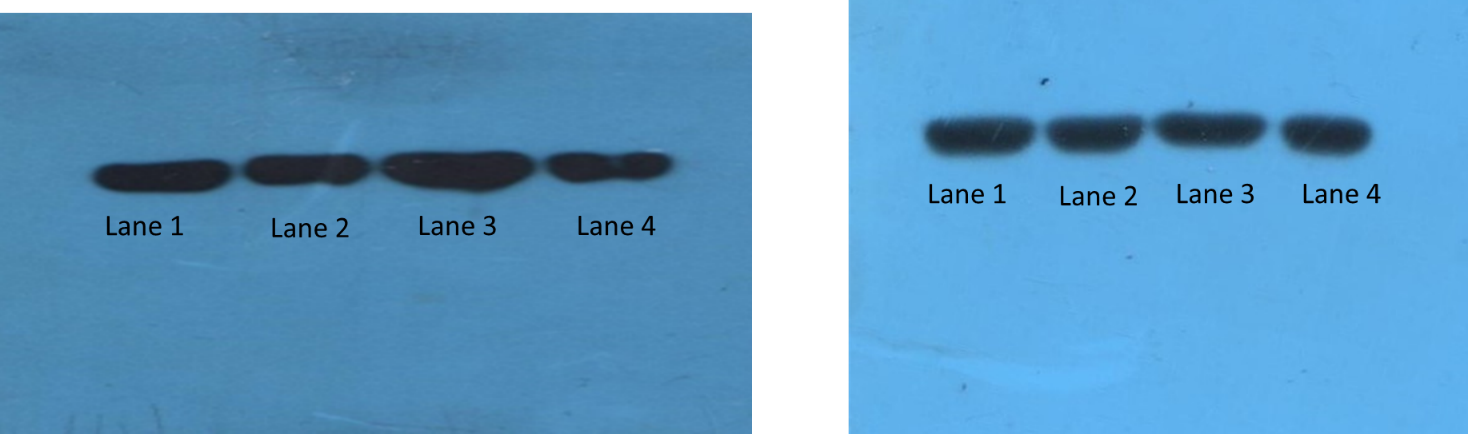


Lane 1: Notch in control group in Prefrontal cortex (PFC)

Lane 2: Notch in Prenatal stress (PNS) group in PFC

Lane 3: Notch in control group in Hippocampus (Hippo)

Lane 4: Notch in PNS group in Hippo

Lane 1: β-actin in control group in Prefrontal cortex (PFC)

Lane 2: β-actin in Prenatal stress (PNS) group in PFC

Lane 3: β-actin in control group in Hippocampus (Hippo)

Lane 4: β-actin in PNS group in Hippo

**d**


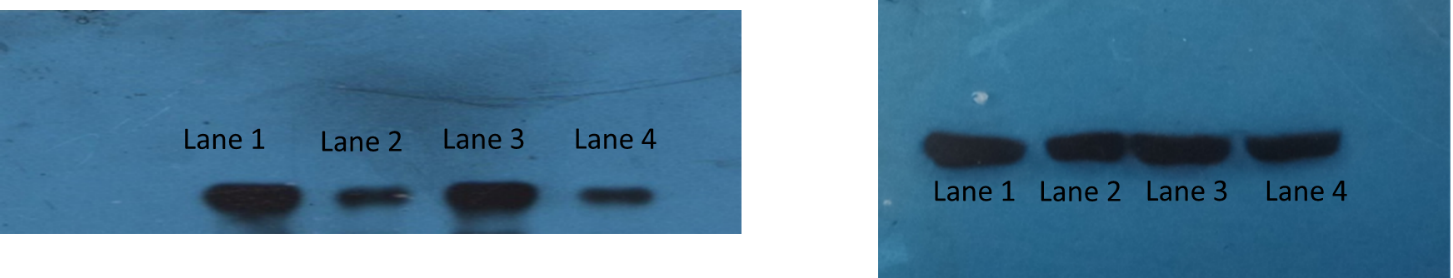


Lane 1: BDNF in control group in Prefrontal cortex (PFC)

Lane 2: BDNF in Prenatal stress (PNS) group in PFC

Lane 3: BDNF in control group in Hippocampus (Hippo)

Lane 4: BDNF in PNS group in Hippo

**e**

Lane 1: β-actin in control group in Prefrontal cortex (PFC)

Lane 2: β-actin in Prenatal stress (PNS) group in PFC

Lane 3: β-actin in control group in Hippocampus (Hippo)

Lane 4: β-actin in PNS group in Hippo
